# Recent advances in the metabolic pathways and microbial production of coenzyme Q

**DOI:** 10.1007/s11274-022-03242-3

**Published:** 2022-02-18

**Authors:** Fabien Pierrel, Arthur Burgardt, Jin-Ho Lee, Ludovic Pelosi, Volker F. Wendisch

**Affiliations:** 1 Univ. Grenoble Alpes, CNRS, UMR 5525, VetAgro Sup, Grenoble INP, TIMC, 38000 Grenoble, France; 2 Genetics of Prokaryotes, Faculty of Biology and Center for Biotechnology (CeBiTec), Bielefeld University, Bielefeld, Germany; 3 Department of Food Science & Biotechnology, Kyungsung University, Busan, South Korea

**Keywords:** Coenzyme Q_10_ (CoQ_10_), *Corynebacterium glutamicum*, *Escherichia coli*, Metabolic engineering, Q complex, Ubi super complex, Yeast

## Abstract

Coenzyme Q (CoQ) serves as an electron carrier in aerobic respiration and has become an interesting target for biotechnological production due to its antioxidative effect and benefits in supplementation to patients with various diseases. Here, we review discovery of the pathway with a particular focus on its superstructuration and regulation, and we summarize the metabolic engineering strategies for overproduction of CoQ by microorganisms. Studies in model microorganisms elucidated the details of CoQ biosynthesis and revealed the existence of multiprotein complexes composed of several enzymes that catalyze consecutive reactions in the CoQ pathways of *Saccharomyces cerevisiae* and *Escherichia coli*. Recent findings indicate that the identity and the total number of proteins involved in CoQ biosynthesis vary between species, which raises interesting questions about the evolution of the pathway and could provide opportunities for easier engineering of CoQ production. For the biotechnological production, so far only microorganisms have been used that naturally synthesize CoQ_10_ or a related CoQ species. CoQ biosynthesis requires the aromatic precursor 4-hydroxybenzoic acid and the prenyl side chain that defines the CoQ species. Up to now, metabolic engineering strategies concentrated on the overproduction of the prenyl side chain as well as fine-tuning the expression of *ubi* genes from the ubiquinone modification pathway, resulting in high CoQ yields. With expanding knowledge about CoQ biosynthesis and exploration of new strategies for strain engineering, microbial CoQ production is expected to improve.

## Introduction

**Fig. 1:**
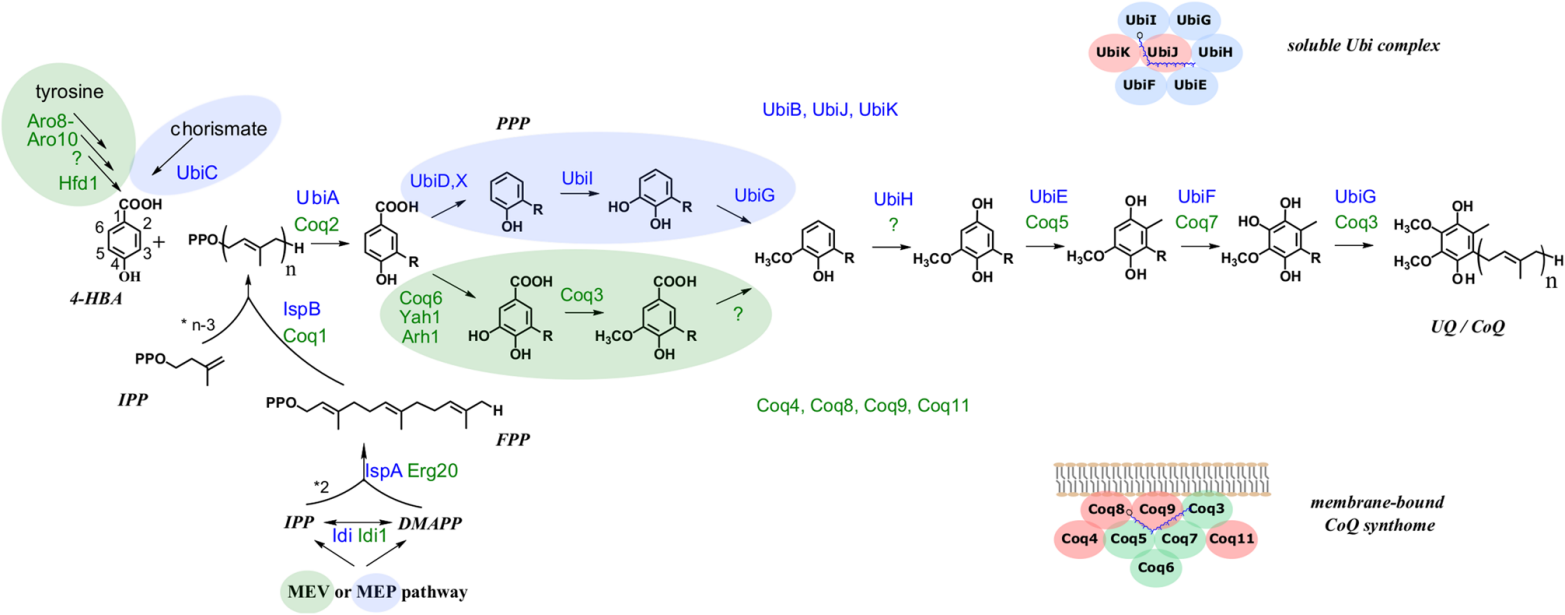
Comparative view of the eukaryotic (*S. cerevisiae*) and prokaryotic (*E. coli*) CoQ/UQ biosynthesis pathways. The proteins are in blue (*E. coli*) or green (*S. cerevisiae*), and the steps that differ between both organisms are highlighted. The numbering of the carbon atoms applied to all intermediates is given for 4-hydroxybenzoic acid (4-HBA) and the polyprenyl chain (n = 6 for *S. cerevisiae,* n = 8 for *E. coli,* n = 10 for CoQ_10_, the CoQ form found in humans) is depicted by R on all intermediates derived from 4-HBA. The Ubi complex and the CoQ synthome illustrate the supramolecular organization of some proteins of the pathways (enzymes in green/blue, accessory proteins in pink). Isopentenyl diphosphate (IPP), dimethylallyl diphosphate (DMAPP) and farnesyl diphosphate (FPP) are building blocks for the synthesis of the polyprenyl diphosphate tail which is added onto 4-HBA by UbiA/Coq2

Coenzyme Q (CoQ), also called ubiquinone, plays an essential role in the respiratory chain of eukaryotes and many prokaryotes. CoQ is composed of a benzoquinone head group conjugated to a polyprenyl chain which length varies between organisms. *Saccharomyces cerevisiae* and *Escherichia coli* produce CoQ_6_ and CoQ_8_, respectively, whereas humans synthesize CoQ_10_ (Fig. 1). The most well-known function of CoQ is to transfer electrons and protons in respiratory chains that sustain bioenergetics. CoQ also acts as a cofactor in uridine biosynthesis, fatty acid oxidation, and for mitochondrial uncoupling proteins. Additionally, CoQ possesses antioxidant and lipid-solubility properties that protect lipids and lipoproteins from oxidative damage (Lee et al. 2012). The roles of CoQ are numerous and have been reviewed recently (Abby et al. 2020; Baschiera et al. 2021; Cirilli et al. 2021).

Although CoQ_10_ is synthesized in human cells and taken up with food, age (Kalén et al. 1989), disease states and use of certain pharmacotherapeutic agents such as statins can lead to CoQ_10_ deficiency (Potgieter et al. 2013). Studies support that dietary supplementation of CoQ_10_, i.a., is beneficial for patients with cardiovascular and neurodegenerative diseases by modulating inflammatory and oxidative DNA damage responses (Yubero-Serrano et al. 2012; Gutierrez-Mariscal et al. 2012), improves symptoms of chronic heart failure, reduces cardiovascular mortality (Mortensen et al. 2014), decreases lead-acetate induced neurotoxicity (Yousef et al. 2019) and potentially slows the functional decline in early Parkinson Disease (Shults 2002). Primary CoQ_10_ deficiency is caused by mutations in genes of the synthetic pathway and may lead to, e.g., infantile encephalomyopathy and ataxia, which can be mitigated by CoQ_10_ supplementation (Quinzii et al. 2006). CoQ_10_ is a highly demanded food supplement, mainly in the form of softgels, capsules, and tablets. To increase its bioavailability, self-emulsified drug delivery systems, nanoemulsions, or cyclodextrin complexes have been developed (Arenas‐Jal et al. 2020). Due to its use as a food supplement and to reduce wrinkles (Žmitek et al. 2017), the industrial production of CoQ_10_ is desired. Although different chemical synthetic approaches have been described (Luo et al. 2017), they suffer from poor tautomer selectivity because they generally yield polyprenyl chains with *cis* and *trans* isomers, whereas the natural CoQ isoforms have an all-*trans* configuration. Thus, microbial bio-production of CoQ_10_ has been developed and in the following, we review CoQ biosynthesis in model microorganisms before focusing on CoQ_10_ production by different bacteria.

## Overview of CoQ biosynthesis pathways in microorganisms

Whereas CoQ is found in almost all eukaryotes, its distribution in bacteria is much more narrow as CoQ is encountered only within the phylum *Proteobacteria* (Schoepp-Cothenet et al. 2013). The global architecture of CoQ biosynthesis is shared between bacteria and eukaryotes as exemplified by the prototypic pathways from the bacterium *E. coli* and the yeast *S. cerevisiae* (Fig. 1). Three stages can be distinguished: the synthesis of the precursor of the benzoquinone head group, the synthesis and conjugation of the polyprenyl side chain, and the sequential modifications of the head group on prenylated intermediates.

### Synthesis of the precursors of the head group of CoQ

4-Hydroxybenzoic acid (4-HBA) serves as a precursor of CoQ in prokaryotes and eukaryotes but is produced differently (Fig. 1). Bacteria possess one of two non-orthologous enzymes (UbiC or XanB2) to catalyze the one-step conversion of chorismate to 4-HBA (Siebert et al. 1994; Zhou et al. 2013). The production of 4-HBA from l-tyrosine in eukaryotes is a multi-step process not fully elucidated, which in *S. cerevisiae* depends at least on aromatic aminotransferases I and II (Aro8, Aro9) and the aldehyde dehydrogenase Hfd1 (Payet et al. 2016; Robinson et al. 2021). 4-Aminobenzoic acid (4-ABA) is also a CoQ precursor in the yeasts *S. cerevisiae* and *Schizosaccharomyces pombe* (Pierrel et al. 2010; Marbois et al. 2010; Nishida et al. 2020), and additional molecules like para-coumarate and resveratrol were also identified as precursors (Xie et al. 2015), although they are likely converted into 4-HBA before entering the CoQ pathway. A recent review on precursors of the benzoquinone head group of CoQ is available for further details (Fernández-Del-Río and Clarke 2021).

### Synthesis of the polyprenyl side chain

Isopentenyl diphosphate (IPP) and dimethylallyl diphosphate (DMAPP) are precursors for the side chain of ubiquinone and are the end products of the mevalonate (MVA) pathway in eukaryotes, archaea and some eubacteria or of the methylerythritol phosphate (MEP) pathway in plants and most bacteria (Pérez-Gil and Rodríguez-Concepción 2013). The MVA pathway from *S. cerevisiae* and the MEP pathway from *E. coli* have been reviewed lately (Kawamukai 2018).

IPP and DMAPP are reversibly isomerized by isopentenyl diphosphate isomerase (Idi/Idi1), and two IPP molecules are added to one DMAPP by a farnesyl diphosphate synthase (IspA/Erg20) to generate farnesyl diphosphate (FPP) with 3 isoprenyl units (Fig. 1). FPP is then extended with sequential additions of IPP molecules by a *trans*-isoprenyl diphosphate synthase (IspB/Coq1). The length of the chain is determined by the size of the pocket which accommodates the growing polyprenyl diphosphate in the enzyme (Nagel et al. 2019).

Finally, the polyprenyl diphosphate chain is added onto 4-HBA by a membrane-bound 4-hydroxybenzoate 3-polyprenyl transferase, UbiA in *E. coli* and the related Coq2 in yeast (Li 2016). Interestingly, *E. coli* UbiA promiscuously accepts polyprenyl diphosphates of different lengths (Okada et al. 1998), as a result of polyprenyl diphosphates gaining access to the active site via an unrestricted lateral portal (Cheng and Li 2014). Catalysis occurs in lipid bilayers and the prenylated 4-HBA products are released into membranes (Cheng and Li 2014; Huang et al. 2014).

### Functionalization of the head group

CoQ is obtained after functionalization of the phenyl ring of polyprenyl 4-HBA via one decarboxylation, three hydroxylation and three methylation steps (Fig. 1). Most steps are catalyzed by enzymes that share homology between eukaryotes and prokaryotes. The biochemistry of CoQ biosynthesis has been reviewed recently in bacteria (Aussel et al. 2014; Abby et al. 2020) and eukaryotes (Kawamukai 2016; Alcázar-Fabra et al. 2016; Stefely and Pagliarini 2017; Wang and Hekimi 2019; Fernández-del-Río and Clarke 2021), thus it will only be briefly discussed here.

### Decarboxylation

In *E. coli*, prenyl 4-HBA is decarboxylated into octaprenylphenol by the UbiD-UbiX system that consists of the 3-octaprenyl-4-hydroxybenzoate decarboxylase UbiD and its associated flavin prenyltransferase UbiX (Fig. 1). UbiX produces the prenylated FMN used as a cofactor by UbiD (Marshall et al. 2017, 2019). Although widely conserved in many bacterial species, the UbiD-UbiX system is absent in some, suggesting that alternative systems exist, as recently proposed for *Xanthomonas campestris* and *Francisella tularensis* (Zhou et al. 2019; Kazemzadeh et al. 2021). In eukaryotes, the C1-decarboxylation step remains genetically and biochemically uncharacterized (Fernández-del-Río and Clarke 2021).

### Hydroxylation reactions

The three hydroxylation reactions required for the biosynthesis of CoQ involve a large repertoire of O_2_-dependent hydroxylases in bacteria. In *E. coli*, three related class A flavoprotein monooxygenases (FMOs), UbiH (octaprenyl-methoxyphenol 1-hydroxylase), UbiI (octaprenylphenol 5-hydroxylase), and UbiF (demethoxyubiquinone 6-hydroxylase) hydroxylate the carbon atoms C1, C5, and C6, respectively (Fig. 1). Other bacterial species contain instead newly identified FMOs, like UbiM and UbiL, which are able to hydroxylate several positions of the head group (Pelosi et al. 2016). Some species contain a carboxylate-bridged diiron hydroxylase named Coq7 (demethoxyubiquinone 6-hydroxylase), which catalyzes a C6-hydroxylation (Stenmark et al. 2001). Overall, the number of CoQ hydroxylases present in bacterial genomes is highly variable (1–4), which suggests a complex evolutionary history (Abby et al. 2020). The situation is even more complex if we consider the newly identified O_2_-independent pathway, which is found in ~ 30% of CoQ synthesizing bacteria and involves two U32 proteins, UbiU and UbiV, as putative O_2_-independent hydroxylases (Pelosi et al. 2019). This pathway could be of interest for industrial production of CoQ under O_2_-limiting conditions.

The composition in CoQ hydroxylases seems more homogenous in eukaryotes with Coq6 (4-hydroxy-3-polyprenylbenzoate 5-hydroxylase), related to bacterial FMOs, catalyzing the C5-hydroxylation and Coq7 hydroxylating C6 (Fig. 1). However, some variation exists since a new FMO has recently been demonstrated to replace Coq7 in land plants, green algae and apicomplexans (Latimer et al. 2021; Xu et al. 2021a). The eukaryotic C1-hydroxylase is not yet known.

### Methylation reactions

The three methylation reactions in the biosynthesis of CoQ are catalyzed by the S-adenosyl-l-methionine (SAM)-dependent UbiG (bifunctional 5-O- and 6-O-methyltransferase) and UbiE (C2-methyltransferase) proteins (Fig. 1), which are homologous to Coq3 and Coq5 in yeast, respectively (Kawamukai 2016). UbiG/Coq3 are needed for both O-methylation reactions of the pathway, while the C-methylation reaction is catalyzed by UbiE/Coq5. Note that UbiE is also involved in the biosynthesis of menaquinone in bacteria (Lee et al. 1997).

### Supramolecular organization of the enzymes that modify the head group

After prenylation by UbiA/Coq2, all biosynthetic intermediates of the CoQ pathway are highly hydrophobic due to their polyprenyl tail, which may complicate substrate accessibility for head group-modifying enzymes. Interestingly, aforementioned hydroxylases and methyltransferases are known to be part of multiprotein complexes termed CoQ synthome in *S. cerevisiae* and Ubi complex in *E. coli* (He et al. 2014; Hajj Chehade et al. 2019). Accessory proteins, important for CoQ biosynthesis, but not involved in the catalysis of specific steps, are also found in those complexes (Fig. 1). The complexes have not been structurally characterized and even the stoichiometry of the proteins is unknown (Stefely and Pagliarini 2017; Wang and Hekimi 2019). So far, only a few direct interactions between specific Ubi or Coq proteins have been confirmed.

In *S. cerevisiae*, the CoQ synthome associates with the inner mitochondrial membrane (IMM) and includes Coq4, Coq8, Coq9 and Coq11, in addition to the Coq3, Coq4, Coq5 and Coq7 enzymes (Kawamukai 2016). The function of Coq4 remains elusive. Coq8 belongs to a family of atypical kinases, namely the UbiB family, and has been proposed to couple ATP hydrolysis to the extraction of CoQ precursors from the IMM and/or to the formation of the CoQ synthome (Reidenbach et al. 2018). Coq9 possesses an amphipathic helix that controls membrane association and the binding of lipids, including CoQ biosynthetic intermediates (Lohman et al. 2019). Moreover, Coq9 physically associates with Coq7 and was therefore suggested to present CoQ intermediates to the enzymes of the CoQ synthome (Lohman et al. 2019). At last, Coq11 is also part of the CoQ synthome and is required for efficient CoQ biosynthesis in yeast, but neither plant nor mammalian orthologs have been identified to date (Allan et al. 2015).

In contrast to the yeast CoQ synthome, the Ubi complex in *E. coli* is soluble and contains the five enzymes (UbiE to UbiI) that catalyze the last six reactions of the pathway (Fig. 1), transforming polyprenyl phenol into CoQ (Hajj Chehade et al. 2019). Two additional proteins, UbiJ and UbiK, are required for efficient CoQ biosynthesis, and are part of the Ubi complex (Fig. 1). UbiJ binds CoQ biosynthetic intermediates via its Sterol Carrier Protein 2 (SCP2) domain (Hajj Chehade et al. 2019) and interacts with UbiK (Loiseau et al. 2017), suggesting that UbiJ might present the head group of the hydrophobic intermediates to Ubi enzymes within the Ubi complex (Hajj Chehade et al. 2019). Interestingly, UbiJ is only required for the O_2_-dependent biosynthesis of CoQ, whereas UbiT participates only in the O_2_-independent biosynthesis of CoQ (Pelosi et al. 2019). Whether UbiT is part or not of the Ubi complex is currently unknown, but UbiT has been proposed to replace UbiJ under anaerobic conditions, since it contains an SCP2 domain and was recently shown to bind polyisoprenoid lipids in *Pseudomonas aeruginosa* (Vo et al. 2020). The UbiD/UbiX decarboxylation system is not part of the Ubi complex, but both proteins are soluble in *E. coli* cell extracts and co-migrate at around 700 kDa (Hajj Chehade et al. 2019), compatible with a UbiD_6_-UbiX_12_ association suggested by their individual 3D-multimeric structures (PDB IDs: 4RHE, 5M1D).

Overall, it appears that most head group-modifying steps of the CoQ biosynthesis pathways are taking place within multiprotein complexes composed of hydroxylases, methyltransferases and lipid-binding proteins that may serve in substrate presentation.

### Regulation of CoQ biosynthesis

Besides CoQ, *E. coli* synthesizes two other isoprenoid quinones, demethyl-menaquinone 8 (DMK_8_) and menaquinone (MK_8_). Dioxygen availability has long been known to influence the composition of the quinone pool, high aeration favoring the accumulation of CoQ_8_ over (D)MK_8_, whereas microaerobic or anaerobic conditions increase the MK_8_ content and decrease CoQ_8_ (Nitzschke and Bettenbrock 2018). The biomass-specific CoQ content of aerobic glucose cultures was found to decrease throughout the exponential phase (Bekker et al. 2007). Consistent with early reports of catabolic repression affecting the CoQ pathway, a 2-fold increase in CoQ_8_ content was obtained by using glycerol instead of glucose as a carbon source (Martínez et al. 2019). This effect may be mediated at least in part by transcriptional regulation since the expression of several genes of the pathway (*ubiA,C,D,X*) was increased in glycerol medium compared to glucose (Martínez et al. 2019 and references therein). A previous report that exposure of *E. coli* to low osmotic pressure dramatically increased CoQ_8_ content has recently been disproven (Tempelhagen et al. 2020).

The regulation of CoQ biosynthesis in eukaryotes has been reviewed lately and is particularly complex in mammals (Villalba and Navas 2021). In *S. cerevisiae*, several mechanisms control CoQ production including the phosphorylation level of several Coq proteins, notably Coq7 (Martín-Montalvo et al. 2013), the regulation of the abundance of Coq5 via the Puf3 RNA-binding protein (Lapointe et al. 2018), the Snf2-dependent splicing of the PTC7 mRNA which encodes a phosphatase (Awad et al. 2017). Interestingly, increasing the mitochondrial methylation capacity by deleting the *cho2* gene encoding a phosphatidylethanolamine *N*-methyltransferase resulted in a five-fold elevation of the cellular CoQ content (Ayer et al. 2021). This last study also identified several other mutants with increased CoQ levels (two- to twelve-fold), opening avenues to elucidate the various pathways and actors that control CoQ biosynthesis.

## Strategies to improve ubiquinone-related production in microorganisms

### Improving precursor supply of benzoquinone ring for ubiquinone production

As an alternative to chemical 4-HBA production from petroleum-derived phenol, bio-based production has been substantiated by extending the shikimate pathway or the tyrosine biosynthetic pathway (Lee and Wendisch 2017). To facilitate the 4-HBA production through the extended shikimate pathway, the gene *ubiC* encoding chorismate-pyruvate lyase (CPL) was expressed in *Klebsiella pneumoniae* (Müller et al. 1995), *E. coli* (Barker and Frost 2001), *Pseudomonas putida* (Yu et al. 2016), *S. cerevisiae* (Krömer et al. 2013), or *Corynebacterium glutamicum* (Kitade et al. 2018). In particular, elaborated strain development was extensively carried out in *C. glutamicum*, which features a high 4-HBA tolerance (Kitade et al. 2018; Kallscheuer and Marienhagen 2018; Purwanto et al. 2018). The engineering strategies are as follows; (1) introduction of feedback-resistant CPL from *E. coli* or *Providencia rustigianii*, (2) blocking of carbon flux to the final reactions of aromatic amino acid biosynthesis, TCA cycle, and/or the quinate/shikimate utilization (QSU) pathway, (3) overexpression of the shikimate pathway genes, (4) increased pools of the precursors phosphoenolpyruvate and erythrose-4-phosphate, (5) reduced accumulation of by-products, including lactate, dihydroxyacetone phosphate, and protocatechuate (PCA), and (6) reduced accumulation of shikimate pathway intermediates, including dehydroshikimate and shikimate. As a consequence, the highest 4-HBA product titer up to about 37 g/L was achieved in a two-stage bioprocess (Kitade et al. 2018). Thus, to provide aromatic precursor for CoQ_10_ biosynthesis, feedback-resistant CPL and AroG from *E. coli* were introduced into a mutant *C. glutamicum*, in which *pobA*, *pcaHG*, and *qsuABD* encoding 4-HBA hydroxylase, PCA dioxygenase, and QSU pathway enzymes (putative shikimate importer, 3-dehydroshikimate dehydratase, and quinate/shikimate dehydrogenase), respectively, were deleted (Burgardt et al. 2021). Meanwhile, *P. taiwanensis* was tailored to produce 4-HBA via l-tyrosine (Lenzen et al. 2019). This was enabled by expressing *tyrA*^fbr^, *tal*, *fcs*, *ech*, and *vdh* coding for a mutated prephenate dehydrogenase, tyrosine-ammonia lyase, feruloyl-CoA synthetase, enoyl-CoA hydratase, and vanillin dehydrogenase, together with blockage of carbon flux to l-tryptophan, homogentisate, and PCA. The resulting strain yielded about 10 g/L 4-HBA from glycerol in fed-batch cultivations. Besides 4-HBA, 4-ABA can also be used as aromatic precursor of CoQ to form 3-hexaprenyl-4-aminobenzoate by the action of 4-HBA-polyprenyl transferase in yeasts (Marbois et al. 2010; Nishida et al. 2020). A feasibility study to produce 4-ABA (around 0.25 mM) via chorismate was implemented in a mutant *S. cerevisiae* by expressing *abz1* encoding 4-aminobenzoate synthase (Krömer et al. 2013). Several microbes such as *E*. *coli* (Huang and Gibson 1970; Koma et al. 2014), *C*. *glutamicum* (Kubota et al. 2016), and *Bacillus subtilis* (Averesch and Rothschild 2019) have been successfully engineered for high titer 4-ABA production. Of these, the highest titer of 4-ABA (about 43 g/L) was obtained by introduction of 4-ABA biosynthetic step from chorismate into the recombinant *C. glutamicum* overexpressing the shikimate pathway genes (Kubota et al. 2016).

### Improving precursor supply of polyprenyl diphosphate for ubiquinone production

Since IPP and DMAPP are building blocks not only for the polyprenyl tail of CoQ, but also for a vast variety of natural terpenes and terpenoids like chlorophylls, carotenoids and various quinones, ways to increase their supply have been studied for many organisms and products.

**Fig. 2:**
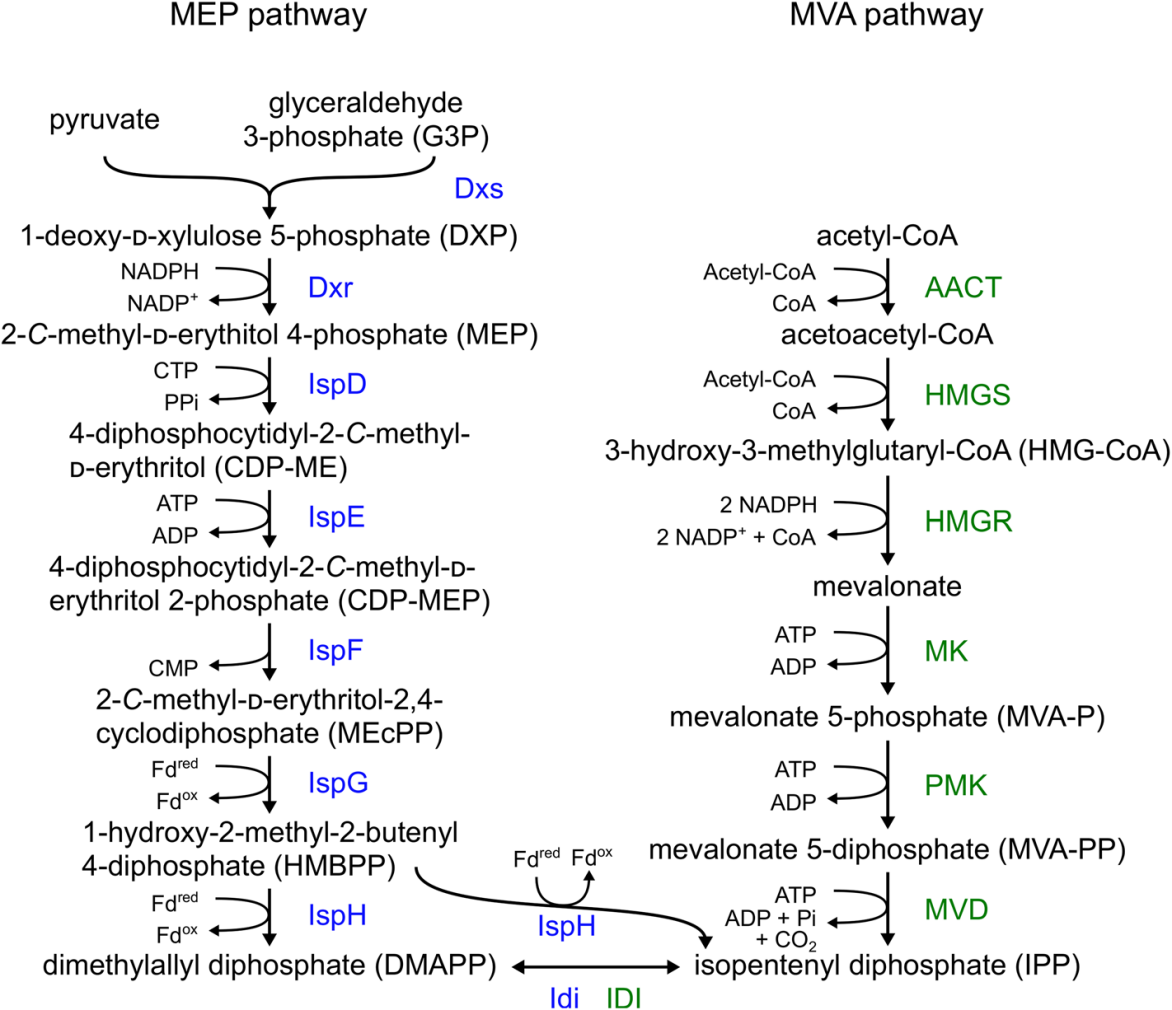
Overview of the MEP and MVA pathways. *E. coli* gene product names in blue represent the reactions of the MEP pathway and enzyme names in green represent the reactions of the MVA pathway in *S. cerevisiae*

The entry point for the MEP pathway is the condensation of glyceraldehyde 3-phosphate (G3P) and pyruvate to 1-deoxy-d-xylulose 5-phosphate (DXP) by DXP synthase (Dxs), followed by reduction to MEP by DXP reductoisomerase (Dxr) (Fig. 2). MEP is converted to IPP in a series of reactions, catalyzed by 2-C-methyl-d-erythritol 4-phosphate cytidylyltransferase (IspD), 4-diphosphocytidyl-2-C-methyl-d-erythritol kinase (IspE), 2-C-methyl-d-erythritol 2,4-cyclodiphosphate synthase (IspF), 4-hydroxy-3-methylbut-2-enyl diphosphate synthase (IspG) and 4-hydroxy-3-methylbut-2-en-1-yl diphosphate reductase (IspH). IPP is isomerized to DMAPP by isopentenyl-diphosphate isomerase (Idi) (Rohmer 1999). Most metabolic engineering strategies to produce IPP-derived terpenes and terpenoids have followed the rationale of overexpressing genes that code for rate-limiting enzymes in the MEP pathway. The overexpression of *dxs* and *idi* has been established in different organisms like *C. glutamicum* and the cyanobacterial *Synechocystis* sp. for the production of patchoulol (Henke et al. 2018) and bisabolene (Rodrigues and Lindberg 2021), respectively. Overexpressing *dxs*, *dxr* and *idi* has improved production of isoprene in *E. coli* (Lv et al. 2013) and menaquinone-7 in *B. subtilis* (Ma et al. 2019; Liao et al. 2021). Volke et al. showed by metabolic control analysis in *E. coli* that indeed Dxs and Idi constitute major flux controlling steps and that upon *dxs* overexpression, 2-C-methyl-d-erythritol 2,4-cyclodiphosphate accumulates intracellularly, while it is also exported outside the cells rather than being reduced to 4-hydroxy-3-methylbut-2-enyl diphosphate. Overexpression of *ispG* and *ispH* did not increase the flux, however (Volke et al. 2019).

Besides the MEP pathway, IPP and DMAPP are synthesized via the MVA pathway that branches off the central carbon metabolism at acetyl-CoA. Two acetyl-CoA molecules are condensed to acetoacetyl-CoA by an acetoacetyl-CoA thiolase (AACT) (Fig. 2). This is followed by another condensation with acetyl-CoA to 3-hydroxy-3-methylglutaryl-CoA (HMG-CoA) via HMG-CoA synthase (HMGS) and subsequent reduction by HMG-CoA reductase (HMGR) to mevalonate. These reactions comprise the upper mevalonate pathway. The remaining reactions, referred to as the lower mevalonate pathway, contain two phosphorylation steps and a decarboxylation by mevalonate kinase (MK), phosphomevalonate kinase (PMK) and mevalonate diphosphate decarboxylase (MVD), respectively, yielding IPP (Miziorko 2011). The utilization of the MVA pathway in addition to the native MEP pathway in *E. coli* has been employed for a variety of products like isoprene (Liu et al. 2019), limonene (Wu et al. 2019), isoprenoid alcohols (Zada et al. 2018) as well as CoQ_10_ (Zahiri et al. 2006). The MVA pathway genes were heterologously expressed and originated from *Streptococcus pneumoniae*, *Enterococcus faealis*, *S. cerevisiae* and *Methanosarcina mazei* amongst others. A synthetic pathway was designed as alternative to the MEP and MVA pathways using a retrosynthetic approach with the goal to challenge the limitations in the natural pathways caused by carbon and energy inefficiencies, complex chemistry and regulatory mechanisms. This pathway centers on the production of isoprenoid alcohols, e.g. prenol or isoprenol, in order to diphosphorylate them to IPP and DMAPP and enabled *E. coli* to produce more than 2 g/L of prenol (Clomburg et al. 2019).

Besides engineering the direct precursor pathways, other approaches have successfully improved production of target compounds. Disruption or downregulation of pathways that compete for the common precursors IPP and DMAPP like carotenoid and hopanoid biosynthesis, has led to higher production of patchoulol by *C. glutamicum* (Henke et al. 2018) and CoQ_10_ by *Rhodobacter sphaeroides* (Zhu et al. 2017a) and *Rhodopseudomonas palustris* (Xu et al. 2021b). Another strategy to obtain higher titers would be to modify pathways of the central carbon metabolism. It is long known that optimizing distribution between G3P and pyruvate, the precursors of the MEP pathway, can increase flux towards the MEP pathway (Farmer and Liao 2001). In addition, combining the Entner–Doudoroff pathway with the MEP pathway increased isoprene titers in *E. coli* three-fold (Liu et al. 2013). With the advent of CRISPRi-mediated repression, fast screening of many target genes among different pathways allows to find suitable candidates to direct flux towards IPP and DMAPP in shorter time as it has been shown for *E. coli* (Tian et al. 2019) and *C. glutamicum* (Göttl et al. 2021). Lastly, cofactor economy plays an important role as some enzymes of the MEP and MVA pathways as well as of the CoQ_10_ synthesis are dependent on NAD(P)H, ATP or SAM. Zhou et al. have increased the NADPH pool by expressing a NADH kinase from *S. cerevisiae*, deleting the NADPH-dependent aldehyde reductase YjgB and overexpressing genes coding for flavodoxin I (*fldA*) and flavodoxin/ferredoxin NADP^+^ reductase (*fpr*) that are known to act as a NADPH-Fpr-FldA reducing system and to activate IspG and IspH (Zhou et al. 2017).

Taken together, the pathways and reactions leading up to IPP and DMAPP offer many possibilities for metabolic engineering approaches. But it is crucial to balance the modifications to avoid metabolic pitfalls that compromise the organisms’ vitality. Strategies like changing NAD(P)H/NAD(P)^+^ ratio or central metabolic pathways often are associated with reduced cell growth and overexpression or heterologous expression of genes in target pathways may perturb regulation to prevent buildup of toxic intermediates (George et al. 2018). Further metabolic engineering strategies to specifically produce CoQ_10_ will be addressed in another section.

### CoQ_10_ production by bacteria natively synthesizing CoQ_10_

Since CoQ_10_ biosynthesis requires many enzymatic steps, and since their reaction mechanisms and regulation are still not fully elucidated, first production approaches were based on native CoQ_10_ producers, mainly *Agrobacterium tumefaciens* and *R. sphaeroides* (Table 1). Unlike secreted products of biotechnological interest such as amino acids (Wendisch 2020), CoQ_10_ is cell-bound, i.e., incorporated into cell membranes. Consequently, biomass production had to be maximized, e.g., by media optimization or mutagenesis, to achieve good CoQ_10_ titers (Yuan et al. 2012). Random mutagenesis and selection with menadione and sodium azide as inhibitors of the respiratory system generated mutants that overcame the growth inhibition with increased CoQ_10_ production. Thus, titers up to 350 mg/L were reached in pH–stat fed-batch fermentations using these classically obtained mutants (Kim et al. 2015). *A. tumefaciens* was used for two up-scaling steps (300 L and 5000 L) and produced CoQ_10_ to a cellular content of 8.54 mg/g DCW and a titer of 458 mg/L (Ha et al. 2007a). CoQ_10_ content and titer were elevated upon controlling the concentration of the carbon substrate sucrose and optimizing pH and dissolved oxygen levels (Ha et al. 2007b). *R. sphaeroides* has been employed for 100 L fermentation in which under phosphate limitation a titer of 1.95 g/L was reached, the highest reported in literature (Zhang et al. 2019). *R. sphaeroides* fermentation has been realized commercially as it also benefits from the fact that CoQ production can operate with non-toxic wastewater (He et al. 2021).

**Table 1:** Representative examples of CoQ_10_ production strategies with natural, mutant, and metabolically engineered hosts

Production host	Key strategies	Titer (mg/L)	Content (mg/g)	Volumetric productivity (mg/L/h)	References
Native CoQ producers and derived mutant strains					
*A. tumefaciens* KCCM 10413	Controlling sucrose concentration, fed-batch cultivation	627	9.25	5.23	Ha et al. (2007a)
*A. tumefaciens* KCCM 10413	Controlling pH and dissolved oxygen, 5000 L fed-batch cultivation	458	8.54	3.82	Ha et al. (2007b)
*A. tumefaciens* 1.2554	Media optimization, mutagenesis, fed-batch cultivation	120	3.86	1.25	Yuan et al. (2012)
*A. tumefaciens* S02-13	Adaptive laboratory evolution with menadione and sodium azide	350	~ 4.2	3.89	Kim et al. (2015)
*R. sphaeroides* KACC 91339P	Optimizing fermentation conditions, 150 L fed-batch cultivation	55	8.12	0.78	Kien et al. (2010)
*R. sphaeroides* Shenzhou6	Mutagenesis using atmospheric and room temperature plasma treatment with vitamin K3 for selection pressure	590	–	5.9	Zou et al. (2019)
*R. sphaeroides* HY01	Phosphate limitation, 100 L fed-batch cultivation	1950	~ 24.4	~ 25.7	Zhang et al. (2019)
Metabolically engineered native CoQ producers					
*E. coli*	Expression of *ddsA* from *A. tumefaciens* and MVA pathway genes from *S. pneumoniae*	–	2.43	–	Zahiri et al. (2006)
*E. coli*	Deletion of *ispB*, expression of *ddsA* from *Sphingomonas baekryungensis*, optimization of cultivation conditions	0.70	0.43	0.10	Martínez et al. (2015)
*R. sphaeroides* 2.4.1	Overexpression of *dxs, dxr, idi*, *ispD* (MEP pathway); overexpression of fused *ubiG* and *ubiE*	138	12.94	2.88	Lu et al. (2015)
*R. sphaeroides* 2.4.1	Overexpression of rate-limiting enzymes, increasing NADH/NAD^+^ ratio and oxygen uptake	600	8.3	6.25	Zhu et al. (2017b)
*R. sphaeroides* 2.4.1	Overexpression of transcriptional repressor *ppsR* to decrease carotenoid synthesis and *crtE* to improve GGPP supply	73	5.67	–	Zhu et al. (2017a)
*R. sphaeroides* 2.4.1	Modifying respiratory chain by deletion of *sdhB*, two-step oxygen supply culture strategy	71	4.59	0.74	Zhang et al. (2018)
*R. sphaeroides* ATCC 17023	Deletion of *fruA* and *fruB*, increasing uptake of glucose via non-PTS by expression of *galP*	78	4.53	1.08	Yang et al. (2021)
*R. palustris* TIE-1	Deletion of *shc* and *crtB* to disrupt carotenoid and hopanoid synthesis, overexpression of *dxs*, *dps*, *ubiA*	3.6	8.2	0.05	Xu et al. (2021b)
Metabolically engineered producers that do not natively synthesize CoQ					
*C. glutamicum* ATCC 13032	Metabolic engineering to produce 4-HBA and DPP, expression of *E. coli* genes from ubiquinone pathway	0.43	0.04	0.004	Burgardt et al. (2021)

### Metabolic engineering strategies for the overproduction of CoQ_10_

Metabolic engineering allows for improving production rationally in native CoQ_10_ producers and for enabling CoQ_10_ production in microorganism that do not possess a native CoQ biosynthesis pathway (Lee et al. 2017). Strategies for (heterologous) overproduction involve the identification and elimination of bottlenecks and flux redistribution in the precursor pathways, use of the MVA pathway in addition to the MEP pathway and/or reducing competitive production of carotenoids as reviewed above. CoQ_10_ has been produced in metabolically engineered eukaryotes and prokaryotes, but as there are less studies about eukaryotic producers and their CoQ_10_ content is not competitive with most bacterial production hosts, the following sections will only focus on the latter.

### Metabolic engineering of *E. coli* to produce CoQ_10_

*E. coli* is a natural CoQ_8_ producer and merely the expression of a heterologous decaprenyl diphosphate synthase is required for CoQ_10_ production since the polyprenyl transferase UbiA promiscuously accepts polyprenyl diphosphates of different lengths (Cheng and Li 2014), as was shown before (Martínez et al. 2015). *E. coli* synthesizes both, menaquinone and ubiquinone, with menaquinone biosynthesis being nonessential under aerobic conditions. Blocking the menaquinone pathway in addition to expression of *dxs* and *ubiA* and supplementation of pyruvate and 4-HBA boosted CoQ_8_ content 4-fold. Growth was not affected under aerobic conditions by the disruption of menaquinone biosynthesis (Xu et al. 2014). CoQ_10_ production by this industrially important organism has received attention some years ago (Table 1) (Zahiri et al. 2006; Cluis et al. 2011), but some natively CoQ_10_ producing bacteria like *R. sphaeroides* proved to be superior hosts for CoQ_10_ production. Nevertheless, a recent example of efficient menaquinone-7 (MK_7_) production to a titer of 1350 mg/L has shown that quinone production by *E. coli* should not be underestimated. This was achieved by optimized heterologous expression of MVA pathway genes and screening several heterologous Idi enzymes to improve IPP supply, overexpression of endogenous and exogenous MK pathway genes and enhancing the flux from chorismate to 1,4-dihydroxy-2-naphthoate, the direct precursor for demethylmenaquinone (Gao et al. 2021).

### Genetic engineering of bacteria that natively produce CoQ_10_

Studies on native CoQ_10_ producers that have been genetically engineered for its overproduction are quite rare with exception of *R. sphaeroides*. This purple photosynthetic bacterium emerged as the most promising organism for CoQ_10_ production in recent years and will therefore be the focus here (Table 1). In one approach, genes that code for enzymes of the aerobic respiration chain were deleted due to relationship between CoQ_10_ synthesis and respiration chain activity. A *R. sphaeroides* mutant defective for succinate dehydrogenase (*sdhB*) overproduced CoQ_10_ under low oxygen conditions, which was exploited in a two-step oxygen supply culture strategy to increase the CoQ_10_ titer from 50 mg/L in the wild type to 71 mg/L in the recombinant strain (Zhang et al. 2018). In another study, deletion of the gene for the only known phosphotransferase system (PTS) in *R. sphaeroides*, PTS^Fru^, combined with heterologous expression of a galactose:H^+^ symporter gene to improve provision with PEP as CoQ_10_ precursor increased the glucose consumption rate by 39% and the biomass concentration by 80% compared to the wild type and the CoQ_10_ titer to 78 mg/L, which was 50% higher than the wild type (Yang et al. 2021). Metabolic bottlenecks in the ubiquinone pathway of *R. sphaeroides* were identified to be UbiE, UbiH, and UbiG. A UbiG-UbiE fusion protein overcame this bottleneck (138 mg/L) despite slightly lower biomass concentration than the wild type (Lu et al. 2015). UbiA was not rate-limiting contrary to observations for *E. coli* and *A. tumefaciens* (Zhang et al. 2007; Cluis et al. 2011). Although not fully understood, heterologous expression of *Vitreoscilla* hemoglobin (*vgb*) slightly improved the titer in this *R. sphaeroides* strain (Lu et al. 2015) to 164 mg/L when the NADH/NAD^+^ ratio was modified as well. While increasing the NADH/NAD^+^ ratio influenced the biomass negatively, expression of *vgb* counteracted this and in combination, growth was superior to the parent strain. A fed-batch fermentation yielded 600 mg/L CoQ_10_ production (Zhu et al. 2017b).

### CoQ_10_ production in bacteria that do not natively produce CoQ

A bacterium natively lacking CoQ biosynthesis has recently been engineered for CoQ_10_ production (Table 1) (Burgardt et al. 2021). Previously, *C. glutamicum* was engineered for high-level production of the aromatic CoQ_10_ precursor 4-HBA (Kitade et al. 2018; Purwanto et al. 2018). Two steps were required to enable CoQ_10_ production by the 4-HBA producing *C. glutamicum* strain. First, overproduction of the prenyl precursor of CoQ_10_, decaprenyl diphosphate (DPP), was achieved by heterologous expression of DPP synthase gene *ddsA* from *Paracoccus denitrificans* (Burgardt et al. 2021). Second, genes for the whole ubiquinone pathway from *E. coli* were expressed and the resulting strain produced 0.43 mg/L (Burgardt et al. 2021). Although the titer was low, this is the first proof-of-concept of producing CoQ_10_ by a microorganism lacking native CoQ biosynthesis. The fact that *C. glutamicum* has been used safely for more than 50 years in fermentative amino acid production, which is operated at a scale of 6 million tons per year (Wendisch 2020), forecasts that optimization of CoQ_10_ production by this bacterium holds large potential. Previous engineering of *C. glutamicum* for high-level production of aromatic compounds including the CoQ_10_ precursor 4-HBA (Lee and Wendisch 2017) as well as for products derived from the MEP pathway (Heider et al. 2014; Henke and Wendisch 2019; Li et al. 2021) provides a sound basis to de-bottleneck transfer of CoQ_10_ biosynthesis from native CoQ_10_ producing microbes to *C. glutamicum* and to gain an in-depth understanding of CoQ_10_ biosynthesis in the respective donor microbes.

## Conclusions and future perspectives

CoQ is a key component in eukaryotic and bacterial cells as it is required for energy generation while also fulfilling numerous other functions. Future research has to fully elucidate CoQ biosynthesis since some parts of CoQ biosynthesis remain uncharacterized, e. g., the C1-decarboxylation and the C1-hydroxylation steps in the aromatic ring modification in eukaryotes. Recent advances, however, have been made in the understanding of the UbiD-UbiX system in bacteria, the diversity of CoQ hydroxylases, and especially, the supramolecular organization of enzymes that finalize the aromatic ring modification towards CoQ. Regarding the latter, the structural characterization and stoichiometry of the involved Ubi or Coq proteins are still missing, but hydroxylases and methyltransferases as well as associated lipid-binding proteins have been identified. In terms of microbial production of CoQ_10_, further research on the rational improvement of CoQ_10_ production is required. Although employment of mutagenized natural CoQ_10_ producers and process optimization led to impressive CoQ_10_ titers, the underlying mechanisms have not been understood. Metabolic engineering will not only enable the use of renewable resources for CoQ_10_ production and improve CoQ_10_ titers and productivities, but rational pathway reconstruction will help to expand the knowledge about the CoQ biosynthesis.

## References

[CR1] Abby SS, Kazemzadeh K, Vragniau C (2020). Advances in bacterial pathways for the biosynthesis of ubiquinone. Biochim Biophys Acta Bioenerg.

[CR2] Alcázar-Fabra M, Navas P, Brea-Calvo G (2016). Coenzyme Q biosynthesis and its role in the respiratory chain structure. Biochim Biophys Acta BBA Bioenerg.

[CR3] Allan CM, Awad AM, Johnson JS (2015). Identification of Coq11, a new coenzyme Q biosynthetic protein in the CoQ-synthome in *Saccharomyces cerevisiae*. J Biol Chem.

[CR4] Arenas-Jal M, Suñé-Negre JM, García-Montoya E (2020). Coenzyme Q10 supplementation: efficacy, safety, and formulation challenges. Compr Rev Food Sci Food Saf.

[CR5] Aussel L, Pierrel F, Loiseau L (2014). Biosynthesis and physiology of coenzyme Q in bacteria. Biochim Biophys Acta.

[CR6] Averesch NJH, Rothschild LJ (2019). Metabolic engineering of *Bacillus subtilis* for production of *para*-aminobenzoic acid—unexpected importance of carbon source is an advantage for space application. Microb Biotechnol.

[CR7] Awad AM, Venkataramanan S, Nag A (2017). Chromatin-remodeling SWI/SNF complex regulates coenzyme Q_6_ synthesis and a metabolic shift to respiration in yeast. J Biol Chem.

[CR8] Ayer A, Fazakerley DJ, Suarna C (2021). Genetic screening reveals phospholipid metabolism as a key regulator of the biosynthesis of the redox-active lipid coenzyme Q. Redox Biol.

[CR9] Barker JL, Frost JW (2001). Microbial synthesis of *p*-hydroxybenzoic acid from glucose. Biotechnol Bioeng.

[CR10] Baschiera E, Sorrentino U, Calderan C (2021). The multiple roles of coenzyme Q in cellular homeostasis and their relevance for the pathogenesis of coenzyme Q deficiency. Free Radic Biol Med.

[CR11] Bekker M, Kramer G, Hartog AF (2007). Changes in the redox state and composition of the quinone pool of *Escherichia coli* during aerobic batch-culture growth. Microbiology.

[CR12] Burgardt A, Moustafa A, Persicke M (2021). Coenzyme Q_10_ biosynthesis established in the non-ubiquinone containing *Corynebacterium glutamicum* by metabolic engineering. Front Bioeng Biotechnol.

[CR13] Cheng W, Li W (2014). Structural insights into ubiquinone biosynthesis in membranes. Science.

[CR14] Cirilli I, Damiani E, Dludla PV (2021). Role of coenzyme Q_10_ in health and disease: an update on the last 10 years (2010–2020). Antioxidants.

[CR15] Clomburg JM, Qian S, Tan Z (2019). The isoprenoid alcohol pathway, a synthetic route for isoprenoid biosynthesis. Proc Natl Acad Sci.

[CR16] Cluis CP, Ekins A, Narcross L (2011). Identification of bottlenecks in *Escherichia coli* engineered for the production of CoQ_10_. Metab Eng.

[CR17] Farmer WR, Liao JC (2001). Precursor balancing for metabolic engineering of lycopene production in *Escherichia coli*. Biotechnol Prog.

[CR18] Fernández-Del-Río L, Clarke CF (2021). Coenzyme Q biosynthesis: an update on the origins of the benzenoid ring and discovery of new ring precursors. Metabolites.

[CR19] Gao Q, Chen H, Wang G (2021). Highly efficient production of menaquinone-7 from glucose by metabolically engineered *Escherichia coli*. ACS Synth Biol.

[CR20] George KW, Thompson MG, Kim J (2018). Integrated analysis of isopentenyl pyrophosphate (IPP) toxicity in isoprenoid-producing *Escherichia coli*. Metab Eng.

[CR21] Göttl VL, Schmitt I, Braun K (2021). CRISPRi-library-guided target identification for engineering carotenoid production by *Corynebacterium glutamicum*. Microorganisms.

[CR22] Gutierrez-Mariscal FM, Perez-Martinez P, Delgado-Lista J (2012). Mediterranean diet supplemented with coenzyme Q10 induces postprandial changes in p53 in response to oxidative DNA damage in elderly subjects. Age.

[CR23] Ha S, Kim S, Seo J (2007). Controlling the sucrose concentration increases coenzyme Q10 production in fed-batch culture of *Agrobacterium tumefaciens*. Appl Microbiol Biotechnol.

[CR24] Ha S-J, Kim S-Y, Seo J-H (2007). Optimization of culture conditions and scale-up to pilot and plant scales for coenzyme Q_10_ production by *Agrobacterium tumefaciens*. Appl Microbiol Biotechnol.

[CR25] Hajj Chehade M, Pelosi L, Fyfe CD (2019). A soluble metabolon synthesizes the isoprenoid lipid ubiquinone. Cell Chem Biol.

[CR26] He CH, Xie LX, Allan CM (2014). Coenzyme Q supplementation or over-expression of the yeast Coq8 putative kinase stabilizes multi-subunit Coq polypeptide complexes in yeast coq null mutants. Biochim Biophys Acta BBA Mol Cell Biol Lipids.

[CR27] He S, Lu H, Zhang G, Ren Z (2021). Production of coenzyme Q_10_ by purple non-sulfur bacteria: current development and future prospect. J Clean Prod.

[CR28] Heider SAE, Peters-Wendisch P, Wendisch VF (2014). Metabolic engineering for the microbial production of carotenoids and related products with a focus on the rare C50 carotenoids. Appl Microbiol Biotechnol.

[CR29] Henke NA, Wendisch VF (2019). Improved astaxanthin production with *Corynebacterium glutamicum* by application of a membrane fusion protein. Mar Drugs.

[CR30] Henke NA, Wichmann J, Baier T (2018). Patchoulol production with metabolically engineered *Corynebacterium glutamicum*. Genes.

[CR31] Huang M, Gibson F (1970). Biosynthesis of 4-aminobenzoate in *Escherichia coli*. J Bacteriol.

[CR32] Huang H, Levin EJ, Liu S (2014). Structure of a membrane-embedded prenyltransferase homologous to UBIAD1. PLoS Biol.

[CR33] Kalén A, Appelkvist E-L, Dallner G (1989). Age-related changes in the lipid compositions of rat and human tissues. Lipids.

[CR34] Kallscheuer N, Marienhagen J (2018). *Corynebacterium glutamicum* as platform for the production of hydroxybenzoic acids. Microb Cell Factories.

[CR35] Kawamukai M (2016). Biosynthesis of coenzyme Q in eukaryotes. Biosci Biotechnol Biochem.

[CR36] Kawamukai M (2018). Biosynthesis and applications of prenylquinones. Biosci Biotechnol Biochem.

[CR37] Kazemzadeh K, Hajj Chehade M, Hourdoir G (2021). The biosynthetic pathway of ubiquinone contributes to pathogenicity of *Francisella novicida*. J Bacteriol.

[CR38] Kien NB, Kong I-S, Lee M-G, Kim JK (2010). Coenzyme Q_10_ production in a 150-l reactor by a mutant strain of *Rhodobacter sphaeroides*. J Ind Microbiol Biotechnol.

[CR39] Kim T-S, Yoo J-H, Kim S-Y (2015). Screening and characterization of an *Agrobacterium tumefaciens* mutant strain producing high level of coenzyme Q_10_. Process Biochem.

[CR40] Kitade Y, Hashimoto R, Suda M (2018). Production of 4-hydroxybenzoic acid by an aerobic growth-arrested bioprocess using metabolically engineered *Corynebacterium glutamicum*. Appl Environ Microbiol.

[CR41] Koma D, Yamanaka H, Moriyoshi K (2014). Production of *p*-Aminobenzoic acid by metabolically engineered *Escherichia coli*. Biosci Biotechnol Biochem.

[CR42] Krömer JO, Nunez-Bernal D, Averesch NJH (2013). Production of aromatics in *Saccharomyces cerevisiae*—a feasibility study. J Biotechnol.

[CR43] Kubota T, Watanabe A, Suda M (2016). Production of *para*-aminobenzoate by genetically engineered *Corynebacterium glutamicum* and non-biological formation of an *N*-glucosyl byproduct. Metab Eng.

[CR44] Lapointe CP, Stefely JA, Jochem A (2018). Multi-omics reveal specific targets of the RNA-binding protein Puf3p and its orchestration of mitochondrial biogenesis. Cell Syst.

[CR45] Latimer S, Keene SA, Stutts LR (2021). A dedicated flavin-dependent monooxygenase catalyzes the hydroxylation of demethoxyubiquinone into ubiquinone (coenzyme Q) in *Arabidopsis*. J Biol Chem.

[CR46] Lee J, Wendisch VF (2017). Biotechnological production of aromatic compounds of the extended shikimate pathway from renewable biomass. J Biotechnol.

[CR47] Lee PT, Hsu AY, Ha HT, Clarke CF (1997). A *C*-methyltransferase involved in both ubiquinone and menaquinone biosynthesis: isolation and identification of the *Escherichia coli ubiE* gene. J Bacteriol.

[CR48] Lee B-J, Lin Y-C, Huang Y-C (2012). The relationship between coenzyme Q10, oxidative stress, and antioxidant enzymes activities and coronary artery disease. Sci World J.

[CR49] Lee SQE, Tan TS, Kawamukai M, Chen ES (2017). Cellular factories for coenzyme Q_10_ production. Microb Cell Factories.

[CR50] Lenzen C, Wynands B, Otto M (2019). High-yield production of 4-hydroxybenzoate from glucose or glycerol by an engineered *Pseudomonas taiwanensis* VLB120. Front Bioeng Biotechnol.

[CR51] Li W (2016). Bringing bioactive compounds into membranes: the UbiA superfamily of intramembrane aromatic prenyltransferases. Trends Biochem Sci.

[CR52] Li C, Swofford CA, Rückert C (2021). Heterologous production of α-Carotene in *Corynebacterium glutamicum* using a multi-copy chromosomal integration method. Bioresour Technol.

[CR53] Liao C, Ayansola H, Ma Y (2021). Advances in enhanced menaquinone-7 production from *Bacillus subtilis*. Front Bioeng Biotechnol.

[CR54] Liu H, Sun Y, Ramos KRM (2013). Combination of Entner-Doudoroff pathway with MEP increases isoprene production in engineered *Escherichia coli*. PLoS ONE.

[CR55] Liu C-L, Bi H-R, Bai Z (2019). Engineering and manipulation of a mevalonate pathway in *Escherichia coli* for isoprene production. Appl Microbiol Biotechnol.

[CR56] Lohman DC, Aydin D, Von Bank HC (2019). An isoprene lipid-binding protein promotes eukaryotic coenzyme Q biosynthesis. Mol Cell.

[CR57] Loiseau L, Fyfe C, Aussel L (2017). The UbiK protein is an accessory factor necessary for bacterial ubiquinone (UQ) biosynthesis and forms a complex with the UQ biogenesis factor UbiJ. J Biol Chem.

[CR58] Lu W, Ye L, Lv X (2015). Identification and elimination of metabolic bottlenecks in the quinone modification pathway for enhanced coenzyme Q_10_ production in *Rhodobacter sphaeroides*. Metab Eng.

[CR59] Luo M, Yang X, Hu J (2017). The synthesis of coenzyme Q10. Curr Org Chem.

[CR60] Lv X, Xu H, Yu H (2013). Significantly enhanced production of isoprene by ordered coexpression of genes *dxs*, *dxr*, and *idi* in *Escherichia coli*. Appl Microbiol Biotechnol.

[CR61] Ma Y, McClure DD, Somerville MV (2019). Metabolic engineering of the MEP pathway in *Bacillus subtilis* for increased biosynthesis of menaquinone-7. ACS Synth Biol.

[CR62] Marbois B, Xie LX, Choi S (2010). *para*-Aminobenzoic acid is a precursor in coenzyme Q_6_ biosynthesis in *Saccharomyces cerevisiae*. J Biol Chem.

[CR63] Marshall SA, Payne KAP, Leys D (2017). The UbiX-UbiD system: the biosynthesis and use of prenylated flavin (prFMN). Arch Biochem Biophys.

[CR64] Marshall SA, Payne KAP, Fisher K (2019). The UbiX flavin prenyltransferase reaction mechanism resembles class I terpene cyclase chemistry. Nat Commun.

[CR65] Martínez I, Méndez C, Berríos J (2015). Batch production of coenzyme Q10 by recombinant *Escherichia coli* containing the decaprenyl diphosphate synthase gene from *Sphingomonas baekryungensis*. J Ind Microbiol Biotechnol.

[CR66] Martínez I, Zelada P, Guevara F (2019). Coenzyme Q production by metabolic engineered *Escherichia coli* strains in defined medium. Bioprocess Biosyst Eng.

[CR67] Martín-Montalvo A, González-Mariscal I, Pomares-Viciana T (2013). The phosphatase Ptc7 induces coenzyme Q biosynthesis by activating the hydroxylase Coq7 in yeast. J Biol Chem.

[CR68] Miziorko HM (2011). Enzymes of the mevalonate pathway of isoprenoid biosynthesis. Arch Biochem Biophys.

[CR69] Mortensen SA, Rosenfeldt F, Kumar A (2014). The effect of coenzyme Q_10_ on morbidity and mortality in chronic heart failure. JACC Heart Fail.

[CR70] Müller R, Wagener A, Schmidt K, Leistner E (1995). Microbial production of specifically ring-^13^C-labelled 4-hydroxybenzoic acid. Appl Microbiol Biotechnol.

[CR71] Nagel R, Schmidt A, Peters RJ (2019). Isoprenyl diphosphate synthases: the chain length determining step in terpene biosynthesis. Planta.

[CR72] Nishida I, Yanai R, Matsuo Y (2020). Benzoic acid inhibits coenzyme Q biosynthesis in *Schizosaccharomyces pombe*. PLoS ONE.

[CR73] Nitzschke A, Bettenbrock K (2018). All three quinone species play distinct roles in ensuring optimal growth under aerobic and fermentative conditions in *E. coli* K12. PLoS ONE.

[CR74] Okada K, Kainou T, Tanaka K (1998). Molecular cloning and mutational analysis of the *ddsA* gene encoding decaprenyl diphosphate synthase from *Gluconobacter suboxydans*. Eur J Biochem.

[CR75] Payet LA, Leroux M, Willison JC (2016). Mechanistic details of early steps in coenzyme Q biosynthesis pathway in yeast. Cell Chem Biol.

[CR76] Pelosi L, Ducluzeau AL, Loiseau L (2016). Evolution of ubiquinone biosynthesis: multiple proteobacterial enzymes with various regioselectivities to catalyze three contiguous aromatic hydroxylation reactions. mSystems.

[CR77] Pelosi L, Vo C-D-T, Abby SS (2019). Ubiquinone biosynthesis over the entire O_2_ range: characterization of a conserved O_2_-independent pathway. Mbio.

[CR78] Pérez-Gil J, Rodríguez-Concepción M (2013). Metabolic plasticity for isoprenoid biosynthesis in bacteria. Biochem J.

[CR79] Pierrel F, Hamelin O, Douki T (2010). Involvement of mitochondrial ferredoxin and para-aminobenzoic acid in yeast coenzyme Q biosynthesis. Chem Biol.

[CR80] Potgieter M, Pretorius E, Pepper MS (2013). Primary and secondary coenzyme Q10 deficiency: the role of therapeutic supplementation. Nutr Rev.

[CR81] Purwanto HS, Kang M, Ferrer L (2018). Rational engineering of the shikimate and related pathways in *Corynebacterium glutamicum* for 4-hydroxybenzoate production. J Biotechnol.

[CR82] Quinzii C, Naini A, Salviati L (2006). A mutation in para-hydroxybenzoate-polyprenyl transferase (COQ2) causes primary coenzyme Q10 deficiency. Am J Hum Genet.

[CR83] Reidenbach AG, Kemmerer ZA, Aydin D (2018). Conserved lipid and small-molecule modulation of COQ8 reveals regulation of the ancient kinase-like UbiB family. Cell Chem Biol.

[CR84] Robinson KP, Jochem A, Johnson SE (2021). Defining intermediates and redundancies in coenzyme Q precursor biosynthesis. J Biol Chem.

[CR85] Rodrigues JS, Lindberg P (2021). Metabolic engineering of *Synechocystis* sp. PCC 6803 for improved bisabolene production. Metab Eng Commun.

[CR86] Rohmer M (1999). The discovery of a mevalonate-independent pathway for isoprenoid biosynthesis in bacteria, algae and higher plants. Nat Prod Rep.

[CR87] Schoepp-Cothenet B, van Lis R, Atteia A (2013). On the universal core of bioenergetics. Biochim Biophys Acta BBA - Bioenerg.

[CR88] Shults CW (2002). Effects of coenzyme Q_10_ in early parkinson disease: evidence of slowing of the functional decline. Arch Neurol.

[CR89] Siebert M, Severin K, Heide L (1994). (1994) Formation of 4-hydroxybenzoate in *Escherichia coli*: characterization of the *ubiC* gene and its encoded enzyme chorismate pyruvate-lyase. Microbiology.

[CR90] Stefely JA, Pagliarini DJ (2017). Biochemistry of mitochondrial coenzyme Q biosynthesis. Trends Biochem Sci.

[CR91] Stenmark P, Grünler J, Mattsson J (2001). A new member of the family of di-iron carboxylate proteins. J Biol Chem.

[CR92] Tempelhagen L, Ayer A, Culham DE (2020). Cultivation at high osmotic pressure confers ubiquinone 8–independent protection of respiration on *Escherichia coli*. J Biol Chem.

[CR93] Tian T, Kang JW, Kang A, Lee TS (2019). Redirecting metabolic flux *via* combinatorial multiplex CRISPRi-mediated repression for isopentenol production in *Escherichia coli*. ACS Synth Biol.

[CR94] Villalba JM, Navas P (2021). Regulation of coenzyme Q biosynthesis pathway in eukaryotes. Free Radic Biol Med.

[CR95] Vo C-D-T, Michaud J, Elsen S (2020). The O_2_-independent pathway of ubiquinone biosynthesis is essential for denitrification in *Pseudomonas aeruginosa*. J Biol Chem.

[CR96] Volke DC, Rohwer J, Fischer R, Jennewein S (2019). Investigation of the methylerythritol 4-phosphate pathway for microbial terpenoid production through metabolic control analysis. Microb Cell Factories.

[CR97] Wang Y, Hekimi S (2019). The complexity of making ubiquinone. Trends Endocrinol Metab.

[CR98] Wendisch VF (2020). Metabolic engineering advances and prospects for amino acid production. Metab Eng.

[CR99] Wu J, Cheng S, Cao J (2019). Systematic optimization of limonene production in engineered *Escherichia coli*. J Agric Food Chem.

[CR100] Xie LX, Williams KJ, He CH (2015). Resveratrol and *para*-coumarate serve as ring precursors for coenzyme Q biosynthesis. J Lipid Res.

[CR101] Xu W, Yang S, Zhao J (2014). Improving coenzyme Q_8_ production in *Escherichia coli* employing multiple strategies. J Ind Microbiol Biotechnol.

[CR102] Xu J-J, Zhang X-F, Jiang Y (2021). A unique flavoenzyme operates in ubiquinone biosynthesis in photosynthesis-related eukaryotes. Sci Adv.

[CR103] Xu W, Ma X, Yao J (2021). Increasing coenzyme Q_10_ yield from *Rhodopseudomonas palustris* by expressing rate-limiting enzymes and blocking carotenoid and hopanoid pathways. Lett Appl Microbiol.

[CR104] Yang Y, Li L, Sun H (2021). Improving CoQ_10_ productivity by strengthening glucose transmembrane of *Rhodobacter sphaeroides*. Microb Cell Factories.

[CR105] Yousef SAO, Fahad AA, Abdel Moneim AE (2019). The neuroprotective role of coenzyme Q10 against lead acetate-induced neurotoxicity is mediated by antioxidant, anti-inflammatory and anti-apoptotic activities. Int J Environ Res Public Health.

[CR106] Yu S, Plan MR, Winter G, Krömer JO (2016). Metabolic engineering of *Pseudomonas putida* KT2440 for the production of *para*-hydroxy benzoic acid. Front Bioeng Biotechnol.

[CR107] Yuan Y, Tian Y, Yue T (2012). Improvement of coenzyme Q10 production: mutagenesis induced by high hydrostatic pressure treatment and optimization of fermentation conditions. J Biomed Biotechnol.

[CR108] Yubero-Serrano EM, Gonzalez-Guardia L, Rangel-Zuñiga O (2012). Mediterranean diet supplemented with coenzyme Q_10_ modifies the expression of proinflammatory and endoplasmic reticulum stress-related genes in elderly men and women. J Gerontol Ser A.

[CR109] Zada B, Wang C, Park J-B (2018). Metabolic engineering of *Escherichia coli* for production of mixed isoprenoid alcohols and their derivatives. Biotechnol Biofuels.

[CR110] Zahiri HS, Yoon SH, Keasling JD (2006). Coenzyme Q_10_ production in recombinant *Escherichia coli* strains engineered with a heterologous decaprenyl diphosphate synthase gene and foreign mevalonate pathway. Metab Eng.

[CR111] Zhang D, Li Z, Wang F (2007). Expression of various genes to enhance ubiquinone metabolic pathway in *Agrobacterium tumefaciens*. Enzyme Microb Technol.

[CR112] Zhang J, Gao D, Cai J (2018). Improving coenzyme Q_10_ yield of *Rhodobacter sphaeroides* via modifying redox respiration chain. Biochem Eng J.

[CR113] Zhang L, Liu L, Wang K-F (2019). Phosphate limitation increases coenzyme Q_10_ production in industrial *Rhodobacter sphaeroides* HY01. Synth Syst Biotechnol.

[CR114] Zhou L, Wang JY, Wu J (2013). The diffusible factor synthase XanB2 is a bifunctional chorismatase that links the shikimate pathway to ubiquinone and xanthomonadins biosynthetic pathways. Mol Microbiol.

[CR115] Zhou J, Yang L, Wang C (2017). Enhanced performance of the methylerythritol phosphate pathway by manipulation of redox reactions relevant to IspC, IspG, and IspH. J Biotechnol.

[CR116] Zhou L, Li M, Wang X-Y (2019). Biosynthesis of Coenzyme Q in the Phytopathogen *Xanthomonas campestris* via a Yeast-Like Pathway. Mol Plant-Microbe Interact.

[CR117] Zhu Y, Lu W, Ye L (2017). Enhanced synthesis of coenzyme Q_10_ by reducing the competitive production of carotenoids in *Rhodobacter sphaeroides*. Biochem Eng J.

[CR118] Zhu Y, Ye L, Chen Z (2017). Synergic regulation of redox potential and oxygen uptake to enhance production of coenzyme Q_10_ in *Rhodobacter sphaeroides*. Enzyme Microb Technol.

[CR119] Žmitek K, Pogačnik T, Mervic L (2017). The effect of dietary intake of coenzyme Q10 on skin parameters and condition: results of a randomised, placebo-controlled, double-blind study: The effect of dietary intake of coenzyme Q10 on skin parameters and condition. BioFactors.

[CR120] Zou R-S, Li S, Zhang L-L (2019). Mutagenesis of *Rhodobacter sphaeroides* using atmospheric and room temperature plasma treatment for efficient production of coenzyme Q10. J Biosci Bioeng.

